# Revealing the Diversity and Complex Relationships of Croatian Olive Germplasm

**DOI:** 10.3390/ijms25063170

**Published:** 2024-03-09

**Authors:** Tatjana Klepo, Đani Benčić, Zlatko Liber, Angjelina Belaj, Frane Strikić, Nives Kević, Zlatko Šatović

**Affiliations:** 1 Centre for Pomology and Vegetable Crops, Croatian Agency for Agriculture and Food, 21210 Solin, Croatia; 2 Department of Pomology, Faculty of Agriculture, University of Zagreb, 10000 Zagreb, Croatia; bencic@agr.hr; 3 Department of Biology, Faculty of Science, University of Zagreb, 10000 Zagreb, Croatia; zlatko.liber@biol.pmf.hr; 4 Centre of Excellence for Biodiversity and Molecular Plant Breeding (CoE CroP-BioDiv), 10000 Zagreb, Croatia; zsatovic@agr.hr; 5 Centro “Alameda del Obispo”, Instituto Andaluz de Investigación y Formación Agraria, Pesquera, Alimentaria y de la Producción Ecológica, IFAPA, 14004 Cordoba, Spain; angjelina.belaj@juntadeandalucia.es; 6 Department of Marine Studies, University of Split, 21000 Split, Croatia; fstrikic@unist.hr; 7 Faculty of Science, University of Split, 21000 Split, Croatia; nkevic@pmfst.hr; 8 Department of Plant Biodiversity, Faculty of Agriculture, University of Zagreb, 10000 Zagreb, Croatia

**Keywords:** *Olea europaea* L., variety, molecular markers, SSR, genetic structure

## Abstract

Olive growing in Croatia has a long tradition and is of great economic and social impact. The present study includes a set of 108 tree samples (88 samples corresponding to 60 presumed cultivars and 20 trees of unnamed ones) collected from 27 groves in the entire olive growing area, and is the most comprehensive survey to be conducted in Croatia. The genetic diversity, relationships, and structures of olive plants were studied using eight microsatellite loci. All loci were polymorphic and revealed a total of 90 alleles. A total of 74 different genotypes were detected that were subjected to further diversity and genetic relationship studies. The Fitch–Margoliash tree and Bayesian analysis of population structure revealed a complex relationship between the identified olive genotypes, which were clustered into three gene pools, indicating different origins of Croatian olive germplasms. Excluding the redundant germplasms, 44 different genotypes among the sampled trees of well-known cultivars and 16 new local germplasms were identified. In addition, we provide the etymology of 46 vernacular names, which confirms that the vast majority of traditional Croatian cultivars have common and widespread names. The results presented herein underline the importance of safeguarding local cultivars and conducting continuous surveys.

## 1. Introduction

In the last three decades, great efforts have been made worldwide to explore, identify, discriminate, classify, and collect the genetic resources of olives (*Olea europaea* L.). The olive is a long-lived, wind-pollinated diploid (2*n* = 2*x* = 46) fruit species [1,2,3,4,5]. Most olive subspecies, such as cultivated (*O. e.* subsp. *europaea* var. *europaea*) and wild olives (*O. e.* subsp. *europaea* var. *sylvestris*), are self-incompatible and require the presence of other cultivars as pollen donors (pollinators), which favours outcrossing [4,6,7]. Wild olives, including genuine wild and feral forms, are spontaneously propagated by seed germination (generative propagation), while cultivated olives are mainly propagated by mist propagation of one-year-old cuttings. Despite the ease of propagation, the exchange of minor and local cultivars between olive growers is mostly based on grafting, as the propagation of these varieties by cuttings in nurseries is scarce.

Olive germplasm is still very rich, and, unlike other fruit species, has not suffered significant genetic erosion [8]. Some authors estimate that there are 2600 different olive cultivars [9,10], and that more than 1200 cultivars are cultivated worldwide [11]. However, this is only the tip of the iceberg, as there are numerous different cultivars with similar names, many cases of synonymy (different names for the same cultivar) and homonymy (same name for different cultivars) [12,13,14], and even unexplored olive germplasms that have yet to be found [13,15,16,17]. These are the main reasons why the exact number of olive cultivars is still under debate. Surveying genetic resources is a dynamic and never-ending process to conserve genetic heritage, as shown by various studies carried out in countries such as Albania [18], Azerbaijan, Turkey [14], France [17], Italy [10,19], Malta [20], Spain [15,21], and Tunis [13,22], as well as studies covering the whole Mediterranean area [12,23,24]. Olive growing in Croatia is of great national importance. Central Dalmatia has traditionally been, and still is, the most important olive growing region in the country, followed by Istria, which has seen a rapid expansion of olive growing in recent decades. One of the most important studies based on morphological observations in the territory of former Yugoslavia was published by Bulić [25], who listed 18 cultivars and 218 names. More recently, Strikić et al. [26] described 46 Croatian olive cultivars under 154 different names. Olive growers are motivated to increase the value of their olive oil by producing oils from local cultivars. They are following the trend of olive growing by producing high-quality extra-virgin olive oil, preferably with the Protected Designation of Origin (PDO) label [27]. The Croatian National List of Varieties contains 28 olive cultivars, of which 16 are of Croatian origin and account for 57.14% of olive nursery production [28]. The estimated number of cultivars grown in Croatia is between 40 and 60 [26,29,30,31], with the cultivar ‘Oblica’ being the most widespread, accounting for more than 65% of olives grown in the country [32], and this has been the case for at least a century [33].

Morphological descriptors are still very important for prospecting studies, cataloguing, and identification, with endocarp characteristics being the most discriminating ones [24,34]. These markers are widely used in olive germplasm management and breeding programmes, although their availability during the season is limited and they are highly dependent on agroecological conditions.

Microsatellite markers (simple sequence repeats; SSR), despite the development of new, high-throughput molecular markers, remain the markers of choice for genetic studies as they are highly polymorphic, numerous, distributed across the genome, and inexpensive [24,35,36,37,38]. The main drawback is the need to optimize data within and between laboratories [23,35,36]. Due to their high diversity, hundreds of different genotypes with a reduced number of well-selected microsatellite loci have been identified [23,24,34,39,40,41]. Following the trends in other olive growing countries, several studies have been conducted in Croatia on the genetic diversity and relationships of olive cultivars using amplified fragment length polymorphism (AFLP) and SSR markers [42,43,44,45,46,47,48,49], which mostly deal with the intracultivar variability and the regional diversity of olives.

This study is part of an ongoing project aimed at the collection, characterisation, and conservation of olive genetic resources in Croatia. It represents the most comprehensive study carried out to date in the country. The use of selected SSR markers made the thorough identification of all tree samples under study, the detection of redundant germplasm, and the elucidation of the complex genetic relationships between the different genotypes possible. Special attention was paid to the synonymy and homonymy of detected and unexplored local germplasms. For the first time, we provide the etymology of 46 vernacular names, as a recent study of the World Olive Germplasm Collection in Cordoba, Spain, highlighted the frequent cases of a common and general approach to the naming of cultivars around the world [12]. Finally, we assess the overall genetic diversity of Croatian olive cultivars and their genetic structures.

## 2. Results

### 2.1. Microsatellite Diversity

One hundred and eight individual tree samples of presumed cultivars and unexplored local germplasm were genotyped using eight microsatellite loci, and a total of 90 alleles were found (Table 1). Fifteen alleles (16.67%) were found in a single sample (private alleles). The number of alleles per locus ranged from 7 (UDO19) to 15 (DCA16 and UDO43), with an average of 11.25. Using the genetic data from 108 samples, the polymorphism information content (PIC) of the microsatellite loci ranged from 0.421 at locus UDO99-019 to 0.810 at locus DCA09. The average PIC value was 0.720. Seven out of eight loci had PIC values of more than 0.70 and can be considered highly informative for the identification and classification of olive cultivars [50]. Moreover, the overall probability of identity (PI) was very low (2.93 × 10^−9^), ruling out the possibility that a matching genotype was due to chance alone.

When studying all the different genotypes identified, including those with scarce allelic differentiation, the values of *H_O_* per loci ranged from 0.514 (UDO99-019) to 0.973 (DCA16 and EMO3), with a mean of 0.867 (Table 1). Slightly lower values were observed for *H_E_* than for *H_O_*, ranging from 0.446 (UDO99-019) to 0.854 (DCA09), with a mean of 0.772.

### 2.2. Cultivar Identification

The pairwise comparison of 108 tree samples of 60 presumed cultivars and 20 tree samples of unexplored (unknown) local germplasms revealed a relatively high level of redundant germplasms. A total of 67 tree samples were classified into 15 redundancy groups (G01–G15; Table S1). The redundant germplasms included: (i) duplicates within the same presumed cultivars (trees sampled in different areas sharing the same genotype), (ii) scarce molecular differentiation (1–3 alleles) within and between different presumed cultivars, and (iii) possible errors in survey. As expected, most of the trees presumably collected as cultivars but sampled in different areas had the same SSR profiles. For example, 10 of the 11 trees of ‘Oblica’ sampled across the country were considered duplicates (the same genotype) or molecular variants. Only the ‘Oblica 04’ sample had a significantly different profile, and was labelled as ‘Oblica Ugljan’.

Molecular variants with minor allelic differentiation (1–3 different alleles) were also detected within the redundant germplasm (including redundancies within and between presumed cultivars) in 14 cases. For example, the samples ‘Karbonaca 1’ and ‘Karbunčela 1’ differed in one, and ‘Karbunčela 2’ in three alleles from the representative cultivar ‘Karbonaca 2’. Taking these and similar cases into account, a total of 74 different multilocus genotypes were identified. A total of 26 presumed cultivars shared SSR profiles and belonged to nine identified cultivars; thus, they were considered as the most likely cases of synonymy (Table 2 and Table S1). In this context, the redundancy group of the cultivar ‘Crnica’ showed the highest number of synonymy cases (‘Buža’, ‘Istarska Crnica’, ‘Plominka’, ‘Verunka’, and ‘Žižulača’). Other interesting synonymies were those of the identified cultivar ‘Slivnjača’ (‘Istrijanka’, ‘Mastrinka’, and ‘Starovjerka’) and those of the identified cultivar ‘Oblica‘ (‘Lumbardeška’ and ‘Slatka’).

Only a small number of samples classified as unknown (4 out of 20) shared the same SSR profiles with the identified cultivars, and these thus represent possible redundancies in the survey (Table S1). It is worth noting that only a few cases (10) of detected redundancies were due to possible errors in prospecting (Table S1). The most obvious possible errors occurred for the samples of ‘Istarska bjelica’, where two samples (‘Istarska bjelica 2’ and ‘Istarska bjelica 3’) differed only in a single allele and were considered to be the same cultivar, while the sample ‘Istarska bjelica 1’ turned out to be identical to the cultivar ‘Drobnica’. The sample ‘Istarska bjelica 1’ is considered as the prospecting error.

**Figure 1:**
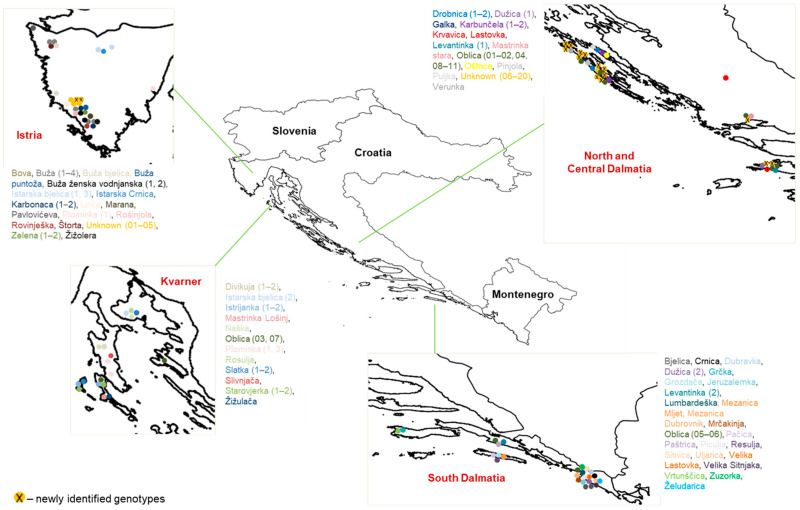
Distribution of olive tree samples collected in Croatia. Each sampled tree is represented by a different colour, and numbers of trees per presumed cultivar are indicated in the parenthesis.

By retaining only one genotype per each redundancy group, the total number of different genotypes identified was 60, of which 44 were identified cultivars and 16 were previously unexplored local germplasms (Figure 1, Table S1). It is interesting to note that most of the 16 new unnamed local genotypes identified in this study originated from traditional olive growing areas, and thus, there was no pressure from cultivar turnover.

Finally, 7 homonymy groups corresponding to 18 different cultivars were also identified (Table 3). Most of the homonymy groups were related to phenotypic traits such as fruit colour (‘Bjelica’ (Croat. bijelo = white) and ‘Crnica’ (Croat. crno = black)), fruit shape ((‘Oblica’ (Croat. oblo = rounded)), or origin (‘Istarska’ and ’Istrijanka’ (Croat. Istarska/Istrijanka = female from Istria) and ‘Mastrinka’ (Croat. mastrinka = wild olive)).

### 2.3. Genetic Relationships and Structure

**Figure 2:**
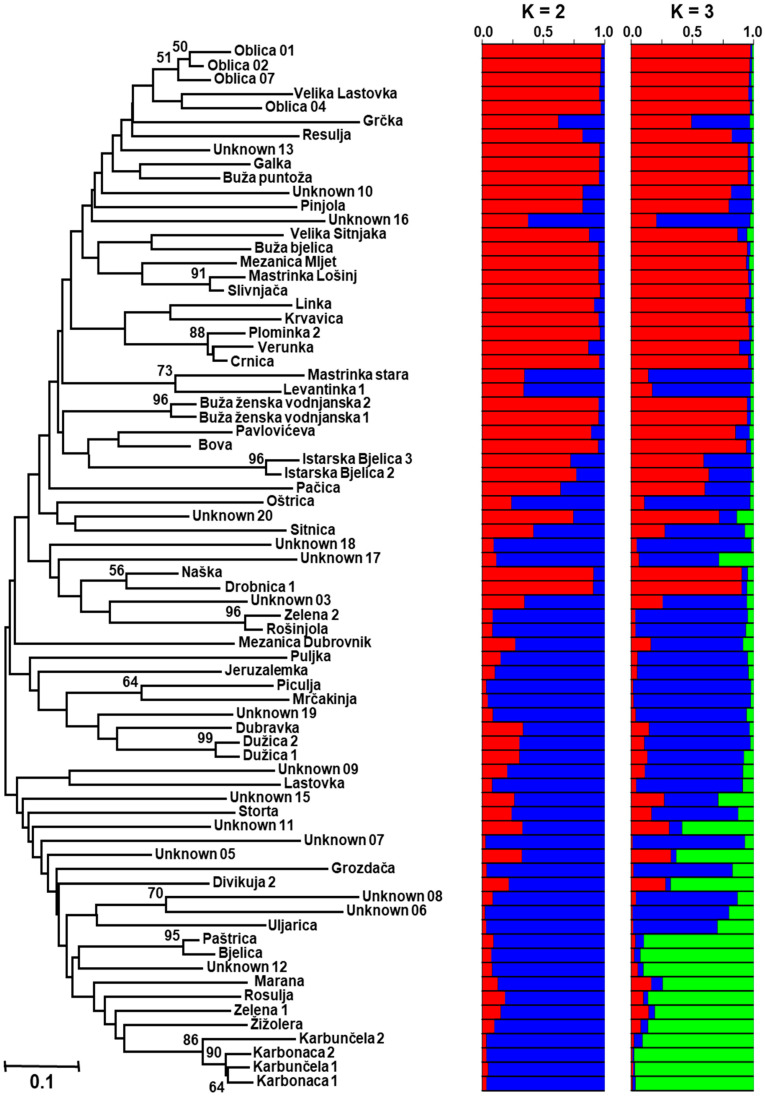
Fitch–Margoliash tree of 74 olive genotypes from Croatia based on eight microsatellite loci. Bootstrap support values greater than 50% of 1000 replicates are given above the branches. Average proportions of membership for K = 2 and 3 gene pools are given as estimated by STRUCTURE. Each genotype is represented by a horizontal box divided into colours. Each colour represents one gene pool (red = gene pool A, blue = gene pool B, and gene pool C = green), and the length of the coloured segment shows the genotype’s estimated proportion of membership in that gene pool.

The unrooted Fitch–Margoliash tree showed the relationships among 74 different multilocus olive genotypes (Figure 2), of which 60 were identified cultivars and 14 were molecular variants.

Three main clusters were identified, and, as expected, the genotypes with low molecular variance were clustered together (Figure 2). The first cluster was the largest and the most mixed in terms of possible geographical origin. It comprised samples of the main Croatian olive cultivar ‘Oblica’, which is thought to originate from Central Dalmatia. The main Istrian cultivar ‘Istarska bjelica’ and one of the main South Dalmatian cultivars ‘Crnica’ were also found in the first cluster. It is worth noting that no close relationship was found in either the samples of ‘Buža’ homonymy (Table 3) or the tree samples of unnamed genotypes within the first cluster. The second cluster consisted of cultivars grown mainly in southern Dalmatia, such as ‘Piculja’ and ‘Dužica’, and only one unnamed sampled tree (Unknown 19), which was found on a farm on the Island of Hvar (Central Dalmatia). In the third cluster, no clear bond was found between geographical origin and Fitch–Margoliash clustering. Very important South Dalmatian cultivars (‘Bjelica’, ‘Lastovka’, and ‘Uljarica’) were clustered with unnamed samples from the Island of Dugi otok (Central Dalmatia) and local cultivars from Istria and Kvarner (‘Žižolera’ and ‘Rosulja’, respectively).

In accordance with the Fitch–Margoliash tree classification, we were able to define the most likely number of gene pools (K = 3) using the STRUCTURE model-based approach to further investigate the underlying genetic structure of the Croatian olive germplasm (Figure 2). The average log-likelihood value of the data as a function of the number of gene pools K, *ln Pr*(X|K), was the highest at K = 3, as was the ΔK value (256.67) (Figure S1). The second-best ΔK value was observed at K = 2 (59.21), while for the remaining hypotheses (K = 4–10), the ΔK values ranged from 0.22 (K = 10) to 6.41 (K = 4). Olive genotypes were probabilistically assigned to inferred gene pools (A, B, or C), or referred to as mixed origin when the probabilities of membership for all gene pools were less than Q < 75%.

Gene pool A consisted of 27 genotypes (36.49%), followed by gene pool B (22 genotypes; 29.73%) and gene pool C (10 genotypes or 13.51%). Fifteen genotypes (20.27%) had a membership probability of less than 75%, and were therefore classified as of mixed origin (AM, BM, or CM).

Twenty-one genotypes (out of 27) of gene pool A had a membership probability of more than 90% (Q_A_ > 0.90). Regarding the geographical origin, most of the genotypes (51.85%) within this gene pool originated from Istria and Kvarner, and had Q_A_ > 0.75. The sample ‘Oblica 01’ could be considered a representative genotype of gene pool A, with Q_A_ = 0.97, although it originated from Central Dalmatia. The largest redundancy group, ‘Crnica’, also had a membership probability of more than 90% (Q_A_ > 0.90). Of the unnamed samples, only two (Unknown 10 and 13) belonged to this gene pool, with a probability of more than 75%. According to STRUCTURE, the main Istrian cultivar ‘Istarska bjelica’ was classified as of mixed origin (AM).

The second genetic gene pool (B) comprised 22 genotypes. Most of them (90.91%) originated from Central and South Dalmatia. In contrast to the previous gene pool A, eight genotypes in gene pool B had a membership probability over 90%, with ‘Piculja’ being the typical genotype, with Q_B_ = 0.96. Six samples were of mixed origin (BM), including ‘Uljarica’. Regarding the gene pool membership of unnamed samples, seven were assigned to gene pool B and three to BM.

Ten genotypes showed a membership probability of more than 75% (Q_C_ > 0.75) for the gene pool C, of which five exceeded 90%, including the most typical genotype, ‘Karbonaca 2’ (Q_C_ = 0.98). Interestingly, equal numbers of Istrian and Kvarner, as well as Dalmatian, genotypes (5 vs. 5) were assigned to this gene pool. Only one unnamed sample (Unknown 12) belonged to this gene pool (Q_C_ = 0.90), while Unknown 05 and Unknown 11 turned out to be of mixed origin (CM).

## 3. Discussion

### 3.1. Microsatellite Diversity

This study represents the most comprehensive survey of olive diversity in the entire Croatian olive growing area. We report here on the use of microsatellite loci for the fingerprinting of olive cultivars and unnamed olive samples. The application of eight microsatellite loci across 108 olive samples revealed rich allelic variation and overall genetic diversity. The microsatellites were polymorphic and had high polymorphism information content (PIC) (above 0.75), except the locus UDO19. Similarly, the low values of probability of identity (PI) confirmed the high usefulness of the selected loci and could be related to the high diversity of Croatian olive germplasms. Additionally, the mean values of observed (*H_O_*) and expected heterozygosity (*H_E_*) were higher than the values previously reported for Croatian [45,47], Croatian and Turkish [42], Azerbaijani and Turkish [14], and Albanian [18] cultivars. These results show the considerable genetic diversity of cultivated germplasms in Croatia. Higher levels of observed (*H_O_*) than expected heterozygosity (*H_E_*) could be the result of the introduction and/or interbreeding of cultivars from different geographical regions (Eastern, Central or Western Mediterranean) [51], followed by selection processes favouring heterozygotes [52].

### 3.2. Cultivar Identification

Selected microsatellites proved their high discriminatory power by identifying 60 different genotypes in a set of 108 tree samples. In previous studies, the estimated numbers of different olive cultivars in Croatia have been between 40 and 60 [26,53].

#### 3.2.1. Synonymy

Complex relationships between olive cultivars and various cases of synonymy revealed by molecular markers have been previously described in numerous studies at the regional [16,19,23,45,54], national [13,15,17,18,20,22,47,55,56,57], and international levels [12,23,58].

The high number of synonyms found for the cultivar ‘Crnica’ (‘Buža’, ‘Istarska Crnica’, ‘Plominka’, ‘Verunka’, and ‘Žižulača’) was not surprising, as ‘Crnica’ is a very generic name (Croat. crno = black). One sample of the cultivar ‘Crnica’ was collected in southern Dalmatia, and its origin in southern Croatia and Montenegro was previously confirmed [43], while the remaining samples were collected in Istria and Kvarner, where they are usually grown [26]. The presence of synonymy cases of a cultivar grown in different areas could be due to the close relations and trade between the southern part (Republic of Dubrovnik, XIV–XIX centuries) and the northern part of today’s Croatia (Istria, which was part of the Republic of Venice from the XV–XVIII centuries), as the cases were obviously named differently. Moreover, similar agroclimatic conditions in the north and south (higher precipitation and greater soil depth) in contrast to the central part of the coast (lack of precipitation, mainly karst soils) could be a main reason why this cultivar is not cultivated in Central Dalmatia.

Other international synonyms confirmed here are ‘Oblica’ and ‘Lumbardeška’. The sample ‘Lumbardeška’ was collected near the town of Dubrovnik (southern Dalmatia), close to the Croatian and Montenegrin state border, and is grown in both countries [25,33,59]. The presence of the same olive cultivars in neighbouring countries seems to be frequent as national borders have changed over time, leading now to numerous international cases of homonymy and synonymy as a consequence of renaming and translation of cultivars from one language to another. This can be found all over the world [12]. The example of the main Croatian cultivar ‘Oblica’, for which more than 30 synonyms have been found in the literature [25,26,33,59,60], shows how complex and crucial the molecular identification of olive cultivars is, although we found only two in this study.

A number of putative cases of synonymy previously reported on the basis of morphological similarity were confirmed here, such as the group ‘Rošinjola’ (‘Rovinješka’ (Croat. ‘Rovinješka’ = female from Rovinj) and ‘Rošinjola’) [28] and ‘Uljarica’ (‘Zuzorka’ and ‘Uljarica’ (Croat. ulje = oil)) [26,60].

The close relationship between the sample pairs ‘Naška’ (Croat. naš = our) and ‘Drobnica’, as well as ‘Paštrica’ and ‘Bjelica’ (Croat. bijelo = white) were also previously reported by Škarica et al. [61] and Slaus-Kantschieder [59], respectively. Two pairs differed in three alleles and one allele, respectively, and were considered synonyms. Together with these, we reported a total of 14 cases of molecular variants between and within cultivars (i.e., inter- and intra-cultivar variability) that could be due to genotyping errors, accumulation of somatic mutations, or the use of highly variable SSRs which are prone to mutations, as previously reported by Trujillo et al. [24]. In any case, we considered these samples as the same genotype.

We also had some obvious examples of survey errors and/or mislabelling as samples of the main Istrian olive cultivar ‘Istarska bjelica’ (Croat. Istarska = Istrian; Croat. bijelo = white). The sample ‘Istarska bjelica 1’ showed the same microsatellite profile as ‘Drobnica’, while the difference between the samples ‘Istarska bjelica 2’ and ‘Istarska bjelica 3’ was only in a single allele. The morphological characteristics (including leaves) and appearance of ‘Drobnica’ and ‘Istarska bjelica’ cultivars are quite different and easy to distinguish [26], and the low intracultivar variability of ‘Istarska bjelica’ (‘Istrska belica’) has already been reported by Lazović et al. [43].

#### 3.2.2. Unexplored Local Germplasm

The unnamed samples analysed here (labelled as Unknown 01–20) showed a high degree of diversity, and only 4 out of 20 were identical to identified cultivars. Sixteen samples that exhibited unique genotypes represent newly discovered local olive genetic resources, suggesting that it is very likely that different genotypes have yet to be found in certain areas. We suspect that these genotypes are minor local cultivars that are still grown in local family farms in neglected agricultural areas such as the Island of Dugi otok (North Dalmatia). Another possibility is that they are feral forms discovered and selected by enthusiastic local farmers who have propagated them vegetatively, as this practise is still common in remote olive growing areas in Croatia. Thus, where the cultivation pressure is lower, a greater diversity could be found, which needs to be evaluated and conserved [12,15,17,20,62]. However, we could not exclude the possibility that these genotypes originate from neighbouring countries such as Italy, Slovenia, and Montenegro.

In any case, urgent ex situ conservation of new olive germplasms and a holistic approach to the evaluation of morphological, phenological, and agronomic traits is needed. Olive growing, like agriculture in general, is facing major challenges due to climate change, which will strongly affect the Mediterranean region. The rich genetic heritage of the species, including known and unexplored local germplasms, feral forms, and wild olives, sheds light on the future of olive cultivation as a reservoir of valuable genes with tolerance and/or resistance to biotic and abiotic stress factors [63,64,65,66].

#### 3.2.3. Homonymy

The existence of numerous very similar or identical generic names of olive cultivars, mostly referring to phenotypic characteristics (e.g., shape, colour) or the origins of the cultivars, has already been reported [12,26]. In fact, a similar phenomenon is noted with Croatian cultivars. Most cultivars have names that refer to typical fruit characteristics, such as fruit colour (Croat. bijelo = white; Croat. crno/karbon/karbun = black; Croat. krv = blood; Croat. zelena = green), fruit size (Croat. sitno = small; Croat. veliko = big;), or fruit shape (Croat. bova = buoy; Croat. dugo = long; Croat. oblo = rounded; Croat. pače = duckling; Croat. pinjol = pine nuts; Croat. želud = acorn; Croat. žižula = jujube (*Ziziphus jujuba* (L.) Gaertn.)).

An excellent example of homonymy are the Istrian olive cultivars ‘Buža’, ‘Buža bjelica’, ‘Buža puntoža’, and ‘Buža ženska vodnjanska’, which show the highest degree of variability. The results of ‘Buža’ homonymy cases support the hypothesis that ‘Buža’ (Croat. dial. buža = hole) is a generic name for a number of different genotypes [46,67,68]. This ambiguity of names is a major problem in the management and conservation of germplasms, nursery production, breeding, and PDO labelling, and it has a negative impact on olive growing in general. A reliable and accurate identification of plant germplasm is a crucial and never-ending work that is necessary for a better knowledge of national and local olive genetic resources [15,17,19,24,54,62]. Great efforts are also being made in order to gain as much knowledge as possible of genetic resources for other fruit species such as apple (*Malus domestica Borkh*.), pear (*Pyrus communis* L.), sweet cherry (*Prunus avium* L.), plum (*Prunus domestica* L.) [69], peach (*Prunus persica* L.) [70], and pistachio (*Pistacia vera* L.) [71].

### 3.3. Genetic Relationships and Structure of Croatian Olive Cultivars

The STRUCTURE analysis revealed three gene pools (A, B, and C), confirming the complexity of the olive patrimony in Croatia. A certain relationship was found between gene pool membership and the geographical origin of cultivars, suggesting a common genetic basis of cultivars and/or frequent exchange of plant material between producers within and between regions [17,42,43].

The main Croatian cultivar ‘Oblica’, represented here with 11 samples from the entire growing area, revealed four different microsatellite profiles that are closely related to each other. In contrast, the identified cultivars of the homonymy group ‘Buža’ were clustered separately on the Fitch–Margoliash tree. However, it was found that they all belonged to gene pool A with high probability (Q_A_ > 0.88). These findings are in partial agreement with previous studies [46] in which samples of ‘Buža’ and ‘Buža puntoža’ were shown to have different DNA profiles, but were clustered together.

Unlike Ercisli et al. [42], we did not find close relationships between ‘Piculja’ (gene pool B) and ‘Istarska bjelica’, nor between ‘Buža’ and ‘Levantinka’. The cultivar ‘Piculja’ (Croat. dial. pica = fruit stone) and the local cultivar ‘Mrčakinja’ (Croat. dia. mrča = myrtle (*Myrtus communis* L.)) are considered synonymous [25,60]. In our study, they showed different DNA profiles and were clustered in gene pool B.

The cultivars of gene pool C could be related to Italian olive cultivars, as ‘Karbonaca’ was probably introduced from the Italian regions of Marche or Lazio [55,59]. Cultivars grown in the Croatian part of the Istrian peninsula, as well as in the Slovenian part of the peninsula, such as ‘Štorta’, have been shown to be closely related to Italian cultivars [55]. The cultivar ‘Uljarica’ was found to be relatively closely related to the Turkish cultivar ‘Ayvalik’ [42]. In this study, both ‘Štorta’ and ‘Uljarica’ showed mixed origins (BM), indicating a common background with foreign cultivars, introgression, and/or exchange of allochthonous material.

In conclusion, the complex pattern of variability, structure, and origin of the Croatian olives presented here underline the importance of continuous survey, collection, and identification of genetic resources, with special attention to the local germplasms. We believe that further genetic studies should also include wild or/and feral olives, which are probably still to be found in limited areas such as the Island of Pag in northern Dalmatia [60]. This completely unknown material is not only a valuable genetic resource of the species [18,54,64,72,73,74], but could provide valuable information on the genetic structure and origin of olive cultivars in Croatia.

## 4. Materials and Methods

### 4.1. Plant Material

With the intention of collecting the most representative autochthonous olive germplasms currently in production (Figure 1, Table S1), a total of 108 trees were sampled in situ and analysed with eight SSRs. The number of sampled trees per presumed cultivar varied from one to eleven. A total of 88 trees belonged to 60 presumed cultivars, while 20 trees represented unnamed local germplasms and were labelled as Unknown 01–20. The information provided by the farmers and the author’s in situ observations including vernacular names for the 60 presumed cultivars, and the geographical data of all sampled trees were recorded. Based on this information and observations, we estimated the ages of the sampled trees, with a minimum age of 20 years. Four to five young and fresh leaves and shoots of the sampled trees used for DNA extraction were collected from 27 olive groves, 15 of which were in Istria and Kvarner, 8 in North and Central Dalmatia and 4 in South Dalmatia. The DNA profiles of known traditional cultivars such as ‘Oblica’, ‘Istarska bjelica’, and ‘Crnica’ were compared and confirmed with profiles available in the literature [24,45,47] and the author’s unpublished data.

### 4.2. DNA Analysis

Genomic DNA was extracted from fresh leaf tissue using a DNeasy Plant Mini Kit (Qiagen^®^, Hilden, Germany). Eight microsatellite (DCA03, DCA09, DCA16, DCA18, EMO03, UDO19, UDO39, UDO43) primer pairs [39,40,41] were used for the analysis. The primers were chosen for their high discriminative power, as shown by Belaj et al. [74], and most of them have been recommended for olive identification [24,35,37]. PCR products were detected using an ABI3730 DNA analyser (Applied Biosystems^®^, Foster City, CA, USA). The results of the detection were analysed using the program package GeneMapper 4.0 (Applied Biosystems^®^).

### 4.3. Data Analysis

For each microsatellite locus, the number of alleles per locus (*N_a_*), the polymorphism information content (PIC), and the probability of identity (PI) were calculated with Cervus v3.0 [75] using the total number of tree samples under study (108). Identical multilocus genotypes were identified using the program GenClone v2.0 [76] and were excluded from the calculation of observed heterozygosity (*H_O_*) and gene diversity (*H_E_*) performed in Cervus.

The proportion-of-shared-alleles distance (*D_psa_*; [77]) between pairs of multilocus genotypes was calculated using MICROSAT [78]. Cluster analysis was performed using the Fitch–Margoliash algorithm [79] as implemented in the FITCH programme of the PHYLIP v3.6b software package [80]. Bootstrap analysis was performed on 1000 bootstrap samples [81].

To infer the genetic structure and determine the number of gene pools, a model-based clustering procedure was applied using the software STRUCTURE v2.3.4 [82]. The Isabella computer cluster at the University Computing Centre (SRCE), University of Zagreb, Croatia, was used to perform thirty runs per gene pool (K = 1 to 11). It consisted of a burn-in period of 200,000 steps followed by 1,000,000 MCMC replicates, assuming an admixture model and correlated allele frequencies. The most likely number of gene pools (K) was chosen by comparing the average estimates of the likelihood of the data, *ln*[Pr(X|K)], for each value of K [82], and by calculating an ad hoc statistic ∆K as implemented in STRUCTURE HARVESTER v0.6.94 [83]. Runs were grouped and averaged using CLUMPAK [84]. Tree samples with membership probabilities of Q < 75% for all gene pools were considered to be of “mixed origin” [85].

## Figures and Tables

**Table 1:** Microsatellite markers used in the study: average number of alleles (*N_a_*), polymorphism information content (PIC), and probability of identity (PI) were calculated based on 108 olive samples, while 74 multilocus genotypes were used to calculate observed (*H_O_*) and expected heterozygosity (*H_E_*).

Reference	Locus	Primer Sequences (5′→3′)	Repeat Motif	Size Range	*N_a_*	PIC	PI	*H_O_*	*H_E_*
[39]	DCA03	CCCAAGCGGAGGTGTATATTGTTAC TGCTTTTGTCGTGTTTGAGATGTTG	(GA)_19_	231–257	9	0.772	0.068	0.946	0.819
[39]	DCA09	AATCAAAGTCTTCCTTCTCATTTCG GATCCTTCCAAAAGTATAACCTCTC	(GA)_23_	162–206	13	0.810	0.050	0.865	0.854
[39]	DCA16	TTAGGTGGGATTCTGTAGATGGTTG TTTTAGGTGAGTTCATAGAATTAGC	(GT)_13_(GA)_29_	122–207	15	0.763	0.070	0.973	0.827
[39]	DCA18	AAGAAAGAAAAAGGCAGAATTAAGC GTTTTCGTCTCTCTACATAAGTGAC	(CA)_4_CT(CA)_3_(GA)_19_	156–197	11	0.740	0.084	0.959	0.817
[40]	EMO3	GGTGTAGCCCAAGCCCTTAT TGCATGACCGTGGTGTAAGT	(CA)_7_	205–218	10	0.796	0.056	0.973	0.838
[41]	UDO99-019	TCCCTTGTAGCCTCGTCTTG GGCCTGATCATCGATACCTC	(GT)_20_(AT)_5_	99–168	7	0.421	0.330	0.514	0.449
[41]	UDO99-039	AATTACCATGGGCAGAGGAG CCCCAAAAGCTCCATTATTGT	(AT)_5_(GT)_11_	106–189	10	0.749	0.081	0.851	0.792
[41]	UDO99-043	TCGGCTTTACAACCCATTTC TGCCAATTATGGGGCTAACT	(GT)_12_	170–224	15	0.708	0.099	0.851	0.782
Mean					11.25	0.720		0.867	0.772
Total					90		2.93 × 10^−9^		

**Table 2:** Synonymy cases identified by means of eight SSR loci: redundancy group, identified cultivar, synonymy group, and the number of different alleles in comparison to the identified cultivar.

Redundancy Group	Identified Cultivar	Synonymy Group	Number of Different Alleles
G01	Crnica	Buža	
		Crnica	
		Istarska Crnica	
		Plominka	
		Verunka	1
		Žižulača	
G02	Oblica	Lumbardeška	
		Oblica	
		Slatka	
G03	Karbonaca	Karbonaca	
		Karbunčela	3
G04	Drobnica	Drobnica	
		Naška	3
G05	Slivnjača	Istrijanka	
		Mastrinka	1
		Slivnjača	
		Starovjerka	
G06	Rošinjola	Rošinjola	
		Rovinješka	
G07	Uljarica	Uljarica	
		Vrtunščica	
		Zuzorka	
G10	Dubravka	Dubravka	
		Želudarica	
G13	Bjelica	Bjelica	
		Paštrica	1

**Table 3:** Cases of homonymy identified using a set of eight SSR loci.

Identified Cultivar	Etymology	Homonymy Group	General Meaning	Number of Different Alleles
Buža	buža (dial.) = hole	Buža	hole	
Buža bjelica	buža (dial.) = hole; bijelo = white			
Buža puntoža	buža (dial.) = hole; punat (dial.) = nipple			
Buža ženska vodnjanska	buža (dial.) = hole; ženska vodnjanska = female from Vodnjan			
Bjelica	bijelo = white	Bjelica	white	
Buža bjelica	buža (dial.) = hole; bijelo = white			
Istarska bjelica	Istarska = Istrian; bijelo = white			
Crnica	crno = black	Crnica	black	
Istarska crnica	Istarska = Istrian; crno = black			
Istarska bjelica	Istarska = Istrian; bijelo = white	Istarska/Istrijanka	female from Istria	
Istarska crnica	Istarska = Istrian; crno = black			
Istrijanka	Istrijanka = female from Istria			
Mastrinka Lošinj	mastrinka (dial.) = wild olive; Lošinj = Lošinj	Mastrinka	wild olive	
Mastrinka stara	mastrinka (dial.) = wild olive; stara = old			
Mezanica Mljet	unknown	Mezanica		
Mezanica Dubrovnik	unknown			
Oblica	oblo = rounded	Oblica	rounded	3
Oblica Ugljan	oblo = rounded; Ugljan = Ugljan			

## Data Availability

The original contributions generated for this study are included in the article/Supplementary Material; further inquiries can be directed to the corresponding author.

## Supplementary Materials

The following supporting information can be downloaded at: https://www.mdpi.com/article/10.3390/ijms25063170/s1.
