# Supplementary figures and images for: Revealing the Diversity and Complex Relationships of Croatian Olive Germplasm
Source: Int J Mol Sci. 2024 Mar 9;25(6):3170. doi: 10.3390/ijms25063170 (PMC10969937; doi:10.3390/ijms25063170)

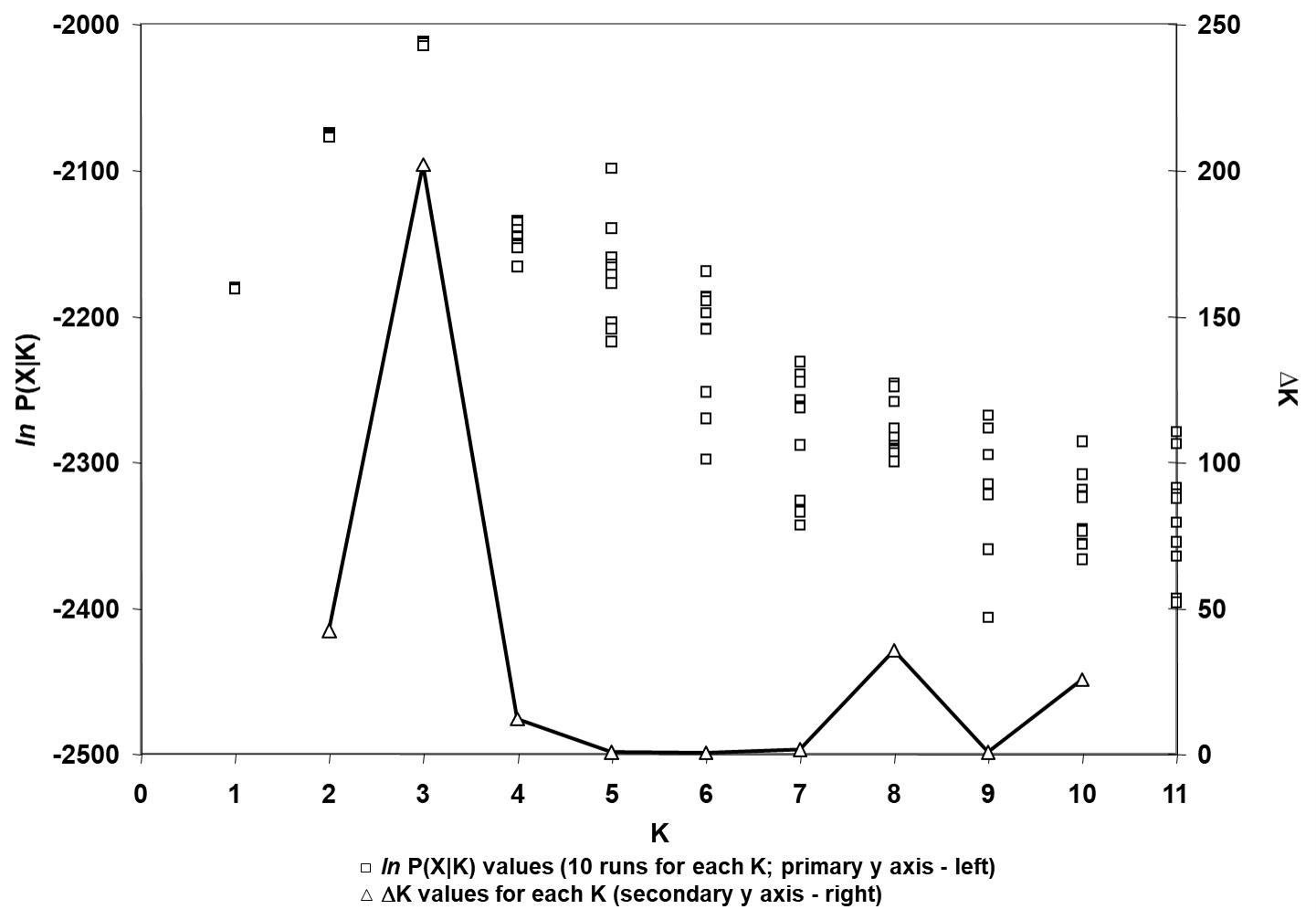

Supplement: Supplementary file 1 [file ijms-25-03170-s001.zip › Figure S1.png]
